# Automated Cognitive Health Assessment Based on Daily Life Functional Activities

**DOI:** 10.1155/2023/5684914

**Published:** 2023-07-07

**Authors:** Shtwai Alsubai, Abdullah Alqahtani, Mohemmed Sha, Sidra Abbas, Michal Gregus, Robert Furda

**Affiliations:** ^1^College of Computer Engineering and Sciences, Prince Sattam Bin Abdulaziz University, AlKharj, Saudi Arabia; ^2^Department of Computer Science, Comsats University, Islamabad, Pakistan; ^3^Information Systems Department, Faculty of Management Comenius University in Bratislava, Odbojárov 10, 82005, Bratislava 25, Slovakia

## Abstract

Dementia is increasing day-by-day in older adults. Many of them are spending their life joyfully due to smart home technologies. Smart homes contain several smart devices which can support living at home. Automated assessment of smart home residents is a significant aspect of smart home technology. Detecting dementia in older adults in the early stage is the basic need of this time. Existing technologies can detect dementia timely but lacks performance. In this paper, we proposed an automated cognitive health assessment approach using machines and deep learning based on daily life activities. To validate our approach, we use CASAS publicly available daily life activities dataset for experiments where residents perform their routine activities in a smart home. We use four machine learning algorithms: decision tree (DT), Naive Bayes (NB), support vector machine (SVM), and multilayer perceptron (MLP). Furthermore, we use deep neural network (DNN) for healthy and dementia classification. Experiments reveal the 96% accuracy using the MLP classifier. This study suggests using machine learning classifiers for better dementia detection, specifically for the dataset which contains real-world data.

## 1. Introduction

Smart homes are a well-known term nowadays and have become a basic need of people. The primary purpose of smart homes is to provide quality life and improve the efficiency in their home [1]. Smart homes provide a more secure and efficient life. Smart homes contain several sensor devices controlled remotely [2]. Smart homes play a vital role in the healthcare industry. Smart homes help medical professionals to monitor patients' health remotely and give urgent care.

The term dementia goes to the loss of cognitive functioning like thinking and remembering with growing age. Dementia individuals cannot control their emotions and personalities and are unable to perform daily base activities [3]. In such conditions, smart homes become a basic need for these people to help them perform daily activities such as cooking, toileting, brushing teeth, getting dressed, watching tv, paying bills, and being social. Smart homes give the confidence to dementia patients to live on their own or with their loved ones. People with dementia are unaware of their mental health [4]. It is necessary to detect dementia early for proper care and mental health treatment [5, 6]. Smart homes are essential in that way for medical professionals. Smart homes contain the Internet of Things (IoT) and sensor base equipment capable of detecting dementia in people automatically. Medical professionals receive data every minute from smart homes and are alert to give treatment when abnormal behavior from older adults [7, 8]. Several techniques have been proposed, such as camera-based assessment, acoustic, voice-based assessment, and robot-based assessment [3]. However, research notices security and privacy issues using these techniques [9–11]. Recent research has proposed AI-based techniques. That has fewer of these issues compared to the abovementioned techniques.

In recently proposed techniques, machine learning techniques are preferred to automatically detect dementia individuals in smart homes. Machine learning classifiers have been categorized as healthy, mild, and dementia individuals [12, 13]. The subject was classified into healthy, mild, and dementia using decision tree, Naïve Bayes, multilayer perceptron, and ensemble AdaBoost [3]. Machine learning uses smart home base generated data as input and gives real-time support to dementia impaired [14, 15].

Real-time activity detection of dementia older adults living in smart homes and performing daily routine activities is essential; several senior older adults do not know about their mental health. Thus, the motivation of the proposed approach is to detect dementia in the early stage and adequately treat people with dementia. Existing studies have limitations for detecting dementia individuals with low detection rates, and we improve the accuracy using the proposed approach [3, 12, 16].

Following are the major contributions of this paper:

- Proposes an approach to classify dementia individuals by analyzing their daily life activities at an early stage using machine learning and deep learning.
- Presents a comparison between machine learning and deep learning algorithms to evaluate the best model and provide a baseline study.
- Deep learning algorithm enhances dementia individuals' detection rate compared to a machine learning algorithm and overperforms the baseline paper detection rate.

The rest of the paper is structured as follows.  Section 2 explains the relevant studies for dementia individual's detection.  Section 3 presents the proposed approach for cognitive dementia detection. Results and discussion are presented in Section 4.  Section 5 provides the discussion on experimental analysis. Finally, Section 5 concludes the paper.

## 2. Literature Review

This section presents past literature studies. Relevant studies on recognizing daily life activities performed by dementia individuals are mentioned below. In the late 1960s idea of smart homes was introduced. One of the most familiar home PCs (ECHO-IV) was made for the accounting family when PCs at home were introduced [17].

Authors in [3] proposed a study to detect cognitively impaired individuals and improve the representation of dementia patients through significant features. They used the ensemble AdaBoost technique to classify the individuals into healthy, mild, and dementia impaired. They used the CASAS dataset. The data was collected from 400 participants. In the dataset, 24 activities were involved, categorized as simple and complex daily life tasks. They achieved 96.02% and 99.6% accuracy compared to other existing techniques. Authors in [12] proposed a machine learning technique to detect the cognitively impaired individual's capability to perform daily base activities to how other individuals perform the same activities. They used the CASAS dataset, which contains 179 volunteers, and performed a complex set of activities in the smart home. They achieved an AUC score of 0.94%.

Author in [18] presented a novel technology integrated health management (TIHM) approach to monitoring dementia patients in their environment. They used machine learning and data analytics techniques to detect the mental health of dementia individuals. They evaluated the efficiency of proposed algorithms by conducting classification.

Author in [16] proposed a machine learning-based approach to observe the performance of dementia individuals in smart homes automatically. To evaluate the proposed approach, they took some older adults to perform activities in a smart home test-bed. They extract features that show how participants perform activities and use them as an input and output in a machine learning algorithm. They assessed that machine learning techniques could differentiate cognitively healthy individuals and individuals with dementia.

The dementia problem that is not identified timely is rising rapidly with the growing population. Author in [19] presented machine learning-based techniques (i. e., support vector machine, logistic regression, artificial neural network, Naive Bayes, decision tree, random forest, and K-nearest neighbor) to detect dementia disease in the early stage. They assessed that support vector machines and random forests accomplish better results on given datasets. Smart and IoT-based technologies are improving the living style of dementia people and can ensure their safety during daily activities. Author in [20] summarized information about activity recognition of dementia individuals using machine learning methods. They merged sensor devices and smart devices during the monitoring process and used warning alarms to prevent abnormal activities in dementia people.

Using the machine learning method, the author in [21] presented a model to detect cognitive and behavioral symptoms of Alzheimer's dementia (AD). The purpose of the proposed model is early detection of mental disorders such as AD, which could alert patients to take action timely. They collected smart home data for 29 older adults, labeled data with activity classes, and extracted ten behavioral features. They used SmoteBOOST and wRACOG algorithms to get reliable results.

The author in [22] presented a robot activity support system (RAS) and explained the role of the smart home. RAS help to perform in-home activities independently. They also collected feedback from those twenty-six individuals who received assistance from RAS in smart homes to evaluate RAS usability. They achieved results of 6.09 out of 7 through the questionnaire. Intelligent robots are performing fully to the betterment of the living style of dementia individuals and help them in social engagement. Author in [23] proposed a proactive listener model to detect AD in older adults that can be implanted in the dialog system of conversational robots. The proposed model classifies user speech into three categories: question, statement, and silence, which generate a particular response. They assessed that they overcome the limitation of speech and breakdown dialogues of dementia individuals with this approach.

To summarize, several research studies [7, 21] exist on daily life activities detection of dementia individuals, but they lack a detection rate. To overcome these limitations, this study proposes an approach to detect dementia individuals by analyzing their daily routine activities at an early stage.

## 3. Proposed Approach

**Figure 1:**
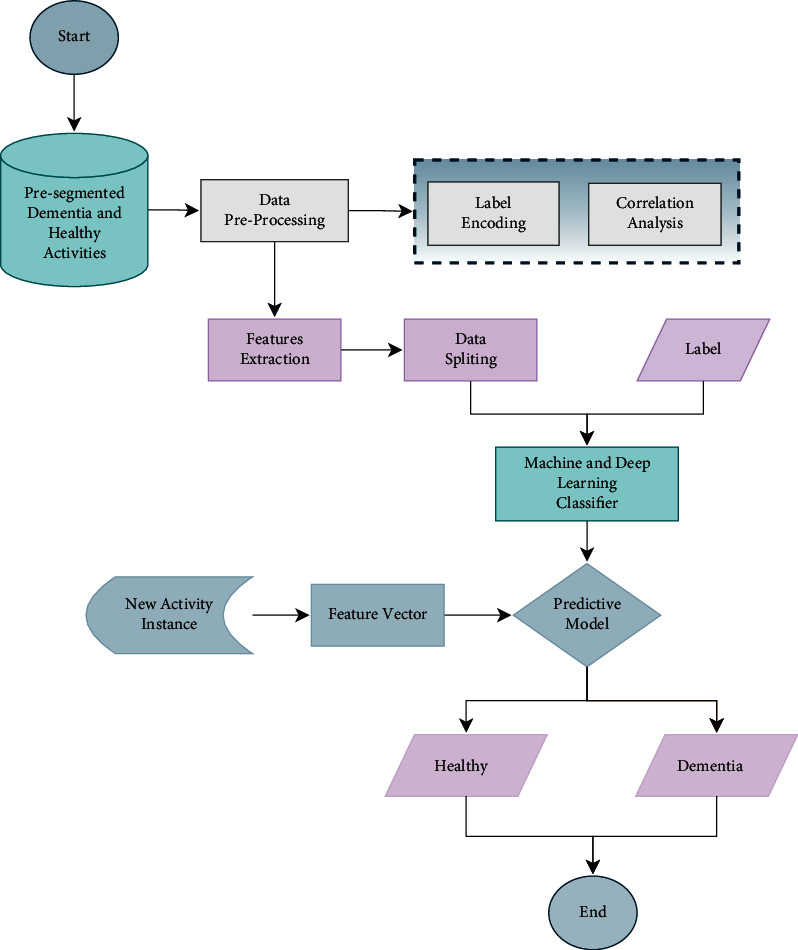
Proposed approach for dementia detection.

The proposed approach focuses on detecting dementia individuals in the early stage. The proposed approach is divided into four steps: data selection, preprocessing, features extraction, and machine learning classifiers to categorize the subject into healthy and dementia individuals. Initially, data was collected from smart home living. Furthermore, features are extracted to detect dementia individuals using the dataset. In the last step, a machine learning and deep learning classifier are trained to detect healthy and dementia individuals.  Figure 1 illustrates the overview of the proposed approach for dementia detection.

### 3.1. Data Selection

We use a subset “Cognitive Assessment Activity (Kyoto)” of publicly available datasets (CASAS) to detect dementia individuals provided by [24]. As per our knowledge, CASAS is the only dataset that automatically detects healthy and dementia individuals using the data collected through simple daily activities in smart homes. The dataset contains 400 individuals, from which 79 individuals (Mean age: 66) divided into 65 healthy and 14 are dementia individuals [3]. They performed daily life activities (i. e., dishes, paying bills, toileting, heating food, watching Tv, and reading newspaper) in smart homes. Data is collected from smart home sensors like motion, force, humidity, door, light, temperature, thermostat, and heat.

### 3.2. Data Preprocessing

Data preprocessing is a procedure that transforms incomplete and inconsistent format data into a well-formed data set. The data preprocessing procedure contains seven steps in machine learning. In the first step, the dataset is acquired and all libraries are imported in the second step. Furthermore, dataset is imported and identified and the missing values are handled . The categorical data in the fifth step is encoded . Furthermore, the dataset is split, and in the last step feature scaling process takes place.

#### 3.2.1. Missing Data Handling

Data handling is a significant step in improving the efficiency of the machine learning model. Frequently, real-life data sets are massive and hold many missing values that can affect the machine learning model's performance. We find a lot of missing values (null or ? Nan) in our data set. We removed some features that provided no information, whereas all features contained zero.

#### 3.2.2. Minmax Scaler

Sometimes machine learning models learn well if features are not scaled. Therefore, it is needed to scale the feature set for efficient model learning. Minmax scaler is the way of data scaling; it shrinks the data within the given range between 0 and 1 without changing the shape of the original distribution.

### 3.3. Features Extraction

The feature extraction step is applied to a raw dataset to convert it into a feature matrix for better understanding. Our dataset contains sensor data collected from a smart home with some dead and irrelevant sensors for simple life activities. To gain better insights and we extract relevant features of daily life activities.

### 3.4. Machine and Deep Learning Classifier

In this section, we present the machine and deep learning algorithms used for healthy and dementia individuals detection. Machine learning and deep learning classifiers are used in various healthcare applications such as cognitive health assessment, tumor detection, breast cancer detection, and lung cancer detection [13, 25, 26]. We use four machine learning algorithms: decision Tree (DT), Naive Bayes (NB), support vector machine (SVM), and multilayer perceptron (MLP). Furthermore, we use deep neural network (DNN) for healthy and dementia classification. DT is a rule-based classification or regression algorithm based on information gain and entropy. It consists of root nodes, decision nodes, and leaf nodes. These nodes are made based on information gain and entropy. Information gain must be the highest among all features to make a feature a root node. This is an iterative process until all features become nodes and reach the leaf node. We set the cross-validation parameter for DT to 10 and confidence to 1.0. We set the batch size to 100 for the NB classifier and kernel estimator to False. For the SVM classifier, we set the batch size to 100, kernel confidence to 1.0, kernel type to poly-kernel, and tolerance parameter to 0.0001. For the MLP classifier, we set the batch size to 100, the learning rate to 0.3, and momentum to 0.2.

We use a tuned deep neural network for cognitive health assessment. We tune various parameters to classify dementia and healthy individuals efficiently. We use five dense layers where in the first dense layer, we set 12 neurons with Relu activation function. In second dense layer, we set 10 neurons with Relu activation function. In third dense layer, we set 8 neurons with Relu activation function. In forth dense layer, we set 6 neurons with Relu activation function. In the fifth dense layer, we set 1 neuron with a sigmoid activation function. A total of 711 trainable parameters are used to train the DNN model on the cognitive health assessment dataset. To compile the DNN model, we use binary cross-entropy with Adam optimizer.

## 4. Experimental Analysis and Results

This section explains our experimental analysis and results through the proposed approach. The purpose of our research work is to detect dementia individuals by evaluating daily life activities. Data splitting is an essential aspect of creating a model based on data. In this way, the performance of the model can evaluate. Usually, train machine learning model data is divided into two parts: training data and testing data. We split the data is 80% as training data and 20% as testing data.

### 4.1. Decision Tree

Table 1 shows the results of decision tree classifier. For healthy individuals, the decision tree achieved a precision of 0.92%, recall of 0.92%, and f1-score of 0.92%. For dementia individuals, the decision tree achieved a precision of 0.91%, recall of 0.92%, and f1-score of 0.92%. Overall it achieved an accuracy of 0.92% for both individuals. It is seen that overall weighted average precision of 0.92%, weighted average recall of 0.92%, and weighted average f1-score of 0.92% for healthy and dementia individuals.

**Figure 2:**
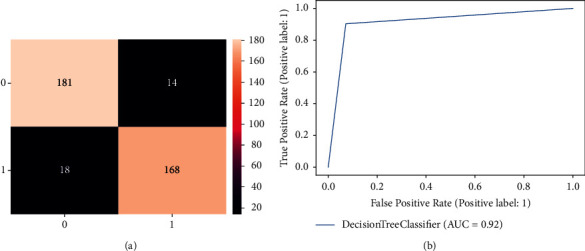
Result of decision tree classifier for dementia detection individuals. (a) Confusion matrix. (b) ROC curve.

Figure 2(a) depicts the confusion matrix of decision tree for detection of dementia individuals. The confusion matrix demonstrates the performance of the classification algorithm. Out of 186 healthy individuals, the decision tree classifies 168 individuals as healthy correctly and incorrectly the remaining 18 as dementia individuals. Furthermore, It is also seen that out of 195 dementia individuals. The decision tree predicts that 14 as healthy and misclassifies the remaining 181 are dementia individuals.  Figure 2(b) shows the ROC curve of the decision tree. Roc curves are frequently used to show a graphical representation of cut-off values. It can be noticed that the ROC curve started increasing from 0.0 to 0.9, and then it became flat.

### 4.2. Naive Bayes

Table 2 represents the results of the Naive Bayes classifier. For healthy individuals, Naive Bayes achieved a precision of 0.63%, recall of 0.92%, and f1-score of 0.75%. For dementia individuals, the decision tree achieved a precision of 0.84%, recall of 0.45%, and f1-score of 0.58%. Overall it achieved an accuracy of 0.69% for both individuals. The table shows the overall average precision of 0.73%, average recall of 0.69%, and average f1-score of 0.67% for healthy and dementia individuals.

**Figure 3:**
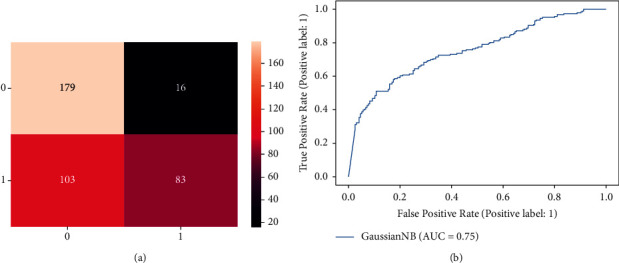
Result of naive bayes classifier for dementia detection individuals. (a) Confusion matrix. (b) ROC curve.

Figure3(a) depicts the confusion matrix of Naive Bayes for detecting dementia individuals. According to this figure, out of 186 healthy individuals, Naive Bayes classifies 83 (63.47%) are healthy correctly and misclassifies the remaining 103 (36.52%) are dementia individuals. Furthermore, It is also shown that out of 195 dementia individuals, Naive Bayes predicts that 179 as dementia correctly and incorrectly, the remaining 16 are dementia individuals. It is noticed that the Naive Bayes classifier obtains low accuracy than the Decision tree.  Figure 3(b) shows the ROC curve of the Naive Bayes. It is shown that the ROC curve started increasing from 0.0 to 0.3. Then it gradually increased to 0.5 and kept increasing to 0.75.

### 4.3. Support Vector Machine (SVM)

Table 3 demonstrates the results of SVM classifier. For healthy individuals, SVM achieved a precision of 0.86%, recall of 0.93%, and f1-score of 0.90%. For dementia individuals, the decision tree achieved a precision of 0.92%, recall of 0.84%, and f1-score of 0.88%. Overall it achieved an accuracy of 0.89% for both individuals. The table shows the overall average precision of 0.89%, average recall of 0.89%, and average f1-score of 0.89% for healthy and dementia individuals.

**Figure 4:**
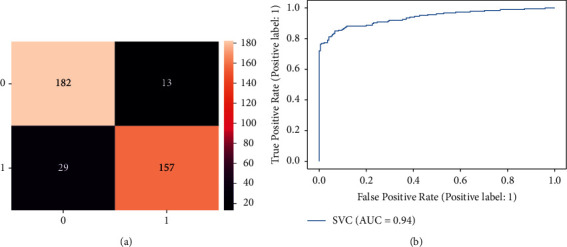
Result of SVM classifier for dementia detection individuals. (a) Confusion matrix. (b) ROC curve.

Figure 4(a) depicts the confusion matrix of SVM for detection of dementia individuals. The graphical representation shows that out of 186 individuals, SVM classifies 157 as healthy correctly and misclassifies the remaining 29 as dementia individuals. Furthermore, SVM predicts that out of 170 dementia individuals, SVM predicts that 182 as dementia correctly and incorrectly, the remaining 13 are healthy individuals. It is noticed that the SVM classifier obtains low accuracy than the Decision tree.  Figure 4(b) shows the ROC curve of the SVM. The ROC curve started increasing from 0.0 to 0.79. Then it gradually increases to 0.94.

### 4.4. Multilayer Perceptron (MLP)

Table 4 shows the results of the decision tree classifier. For healthy individuals, MLP achieved a precision of 0.95%, recall of 0.98%, and f1-score of 0.96%. For dementia individuals, MLP achieved a precision of 0.81%, recall of 0.94%, and f1-score of 0.96%. Overall it achieved an accuracy of 0.96% for both individuals. It is seen that overall weighted average precision of 0.96%, weighted average recall of 0.96%, and weighted average f1-score of 0.96% for healthy and dementia individuals.

**Figure 5:**
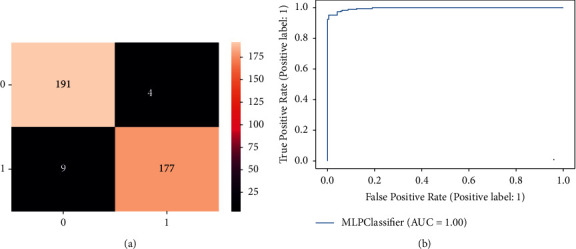
Result of MLP classifier for dementia detection individuals. (a) Confusion matrix (b) Roc curve.

Figure 5(a) depicts the confusion matrix of MLP for detection of dementia individuals. As per this figure, out of 186 healthy individuals, MLP classifies 177 as healthy correctly and misclassifies the remaining 9 as dementia individuals. Furthermore, It is also noticed that out of 195 dementia individuals, MLP predicts that 191 as dementia correctly and the remaining 4 as healthy individuals incorrectly. MLP achieved high accuracy than decision tree, Naive Bayes, and SVM classifiers.  Figure 5(b) shows the ROC curve of the MLP. It is shown that the ROC curve started increasing from 0.0 to 0.79. Then it gradually increases to 0.99.

### 4.5. Deep Neural Network

**Figure 6:**
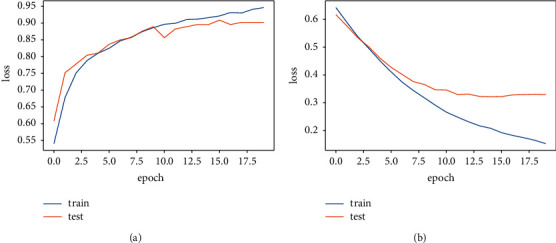
Result of DNN classifier for dementia detection individuals. (a) Accuracy curve (b) loss curve.

Figure 6 shows the accuracy and loss curve.  Figure 6(a) represents the accuracy curve where the blue line shows the training accuracy curve and the orange line shows the testing accuracy curve. Training accuracy started from 0.55% at 0^*th*^ epoch and increased to 0.84 at 1^*st*^ epoch. After that, it increases 1% at each epoch ending on 0.95 at the 18^*th*^ epoch. Testing accuracy started from 0.65% at 0^*th*^ epoch and increased to 0.87 at 1^*st*^ epoch. After that, it started to increase slightly and decreased at each epoch ending at 0.89 at the 18th epoch.  Figure 6(b) represents the loss curve. Training loss started from 0.7 at 0^*th*^ epoch and decreased to 0.39 at 3^*r*  *d*^ epoch. After that, it started decreasing with each epoch ending at 0.15 at 18^*th*^ epoch. Testing loss started from 0.69 at 0^*th*^ epoch and decreased to 0.41 at 2.9^*th*^ epoch. Furthermore, a slight fluctuation at each epoch ends at 0.39 at 18^*th*^ epoch.

## 5. Discussion

This paper proposed an approach to detecting dementia using machine learning (decision Tree, Naive Bayes, SVM, and MLP) and deep learning algorithms. We use CASAS simple daily life activities dataset for experiments. The dataset consists of 1903 instances where healthy individuals are 976 and dementia individuals are 927. We used 80% data as training and 20% as testing. It is noticed that machine learning classifier perform well on data set in the compassion of deep learning. Specifically, the MLP classifier outperforms all other classifiers. It is expected that real-world datasets are small. Our study suggests using a machine learning classifier to get better performance in detecting dementia individuals.

## 6. Conclusion

This paper proposed a machine learning and deep learning-based approach to detect dementia in people. This approach focused on the automated detection of dementia individuals living in smart homes. Our experimental results show that machine learning classifiers give better accuracy than deep learning algorithms using the CASAS dataset. Our study achieved 0.96% accuracy through the MLP classifier compared to other classifiers. In future researchers can collect more real-life dementia individuals for better accuracy.

## Figures and Tables

**Table 1:** Results of decision tree classifier for dementia detection individuals.

	Precision	Recall	F1-score	Support
Healthy	0.92	0.92	0.92	195
Dementia	0.91	0.92	0.92	186
Accuracy	—	—	0.92	381
Weighted avg	0.92	0.92	0.92	381

**Table 2:** Results of naive bayes classifier for dementia detection individuals.

	Precision	Recall	F1-score	Support
Healthy	0.63	0.92	0.75	195
Dementia	0.84	0.45	0.58	186
Accuracy	—	—	0.69	381
Weighted avg	0.73	0.69	0.67	381

**Table 3:** Results of SVM classifier for dementia detection individuals.

	Precision	Recall	F1-score	Support
Healthy	0.86	0.93	0.90	195
Dementia	0.92	0.84	0.88	186
Accuracy	—	—	0.89	381
Weighted avg	0.89	0.89	0.89	381

**Table 4:** Results of MLP classifier for dementia detection individuals.

	Precision	Recall	F1-score	Support
Healthy	0.95	0.98	0.96	195
Dementia	0.98	0.94	0.96	186
Accuracy	—	—	0.96	381
Macro avg	0.96	0.96	0.96	381
Weighted avg	0.96	0.96	0.96	381

## Data Availability

The [Cognitive health assessment] data used to support the findings of this study are included in the article.

## References

[1] Pliatsikas P., Economides A. A. (2022). Factors influencing intention of Greek consumers to use smart home technology. *Applied System Innovation*.

[2] Li W., Yigitcanlar T., Erol I., Liu A. (2021). Motivations, barriers and risks of smart home adoption: from systematic literature review to conceptual framework. *Energy Research & Social Science*.

[3] Javed A. R., Fahad L. G., Farhan A. A. (2021). Automated cognitive health assessment in smart homes using machine learning. *Sustainable Cities and Society*.

[4] Sarkar J. L., Majumder A., Majumder A. (2022). I-health: sdn-based fog architecture for iiot applications in healthcare. *IEEE/ACM Transactions on Computational Biology and Bioinformatics*.

[5] Gillani L. F., Rajarajan M. Anomalies detection in smart-home activities.

[6] Wang W., Liu H., Li J., Nie H., Wang X. (2020). Using cfw-net deep learning models for x-ray images to detect covid-19 patients. *International Journal of Computational Intelligence Systems*.

[7] Fahad L. G., Tahir S. F. (2021). Activity recognition and anomaly detection in smart homes. *Neurocomputing*.

[8] Zhou S., Tan B. (2020). Electrocardiogram soft computing using hybrid deep learning cnn-elm. *Applied Soft Computing*.

[9] Xiong H., Jin C., Alazab M. (2022). On the design of blockchain-based ecdsa with fault-tolerant batch verification protocol for blockchain-enabled iomt. *IEEE journal of biomedical and health informatics*.

[10] Wang W., Chen Q., Yin Z. (2021). Blockchain and puf-based lightweight authentication protocol for wireless medical sensor networks. *IEEE Internet of Things Journal*.

[11] Akram F., Liu D., Zhao P., Kryvinska N., Abbas S., Rizwan M. (2021). Trustworthy intrusion detection in e-healthcare systems. *Frontiers in Public Health*.

[12] Dawadi P. N., Cook D. J., Schmitter M. E. (2013). Automated cognitive health assessment using smart home monitoring of complex tasks. *IEEE transactions on systems, man, and cybernetics: Systems*.

[13] Javed A. R, Sarwar M. U., Beg M. O., Asim M., Baker T., Tawfik H. (2020). A collaborative healthcare framework for shared healthcare plan with ambient intelligence. *Human-centric Computing and Information Sciences*.

[14] Javed A. R, Sarwar M. U., Rehman S., Khan H. U., Otaibi Y. D. A., Alnumay W. S. (2021). Pp-spa: privacy preserved smartphone-based personal assistant to improve routine life functioning of cognitive impaired individuals. *Neural Processing Letters*.

[15] Lee S., Park D. (2021). Adaptive ecg signal compression method based on look-ahead linear approximation for ultra long-term operating of healthcare iot devices (sci). *Hum. Centric Comput. Inf. Sci*.

[16] Dawadi P., Cook D., Parsey C., Schmitter M. E., Schneider M. An approach to cognitive assessment in smart home.

[17] Gotkin K. (2014). When computers were amateur. *IEEE Annals of the History of Computing*.

[18] Enshaeifar S., Zoha A., Markides A. (2018). Health management and pattern analysis of daily living activities of people with dementia using in-home sensors and machine learning techniques. *PLoS One*.

[19] Miah Y., Chowdhury N. E. P., Sharmeen J. S., Mahmud M., Kaiser M. S. (2021). *Performance comparison of machine learning techniques in identifying dementia from open access clinical datasets Advances on Smart and Soft Computing*.

[20] Fikry M., Hamdhana D., Lago P., Inoue S. (2021). *Activity recognition for assisting people with dementia Contactless Human Activity Analysis*.

[21] Alberdi A., Weakley A., Schmitter-Edgecombe M. (2018). Smart home-based prediction of multidomain symptoms related to alzheimer’s disease. *IEEE journal of biomedical and health informatics*.

[22] Wilson G., Pereyda C., Raghunath N. (2019). Robot-enabled support of daily activities in smart home environments. *Cognitive Systems Research*.

[23] Li Y., Lai C., Lala D., Inoue K., Kawahara T. Alzheimer’s Dementia Detection through Spontaneous Dialogue with Proactive Robotic Listeners.

[24] Center for advanced studies (2013). Adaptive Systems (CASAS). “Cognitive assessment activity (kyoto). http://casas.wsu.edu/datasets/.

[25] Mohiyuddin A., Basharat A., Ghani U. (2022). Breast tumor detection and classification in mammogram images using modified yolov5 network. *Computational and Mathematical Methods in Medicine*.

[26] Mehmood M., Rizwan M., Gregus ml M., Abbas S. (2021). Machine learning assisted cervical cancer detection. *Frontiers in Public Health*.

